# Case report: Long-term partial response of apatinib plus paclitaxel as second-line therapy in a patient with metastatic gastric cancer

**DOI:** 10.3389/fphar.2022.888106

**Published:** 2022-08-10

**Authors:** Shengya Fu, Linjuan Li, Xiaofen Li, Qiang Wu, Xiaohui Wang, Yan Huang, Haoyue Hu, Dan Cao

**Affiliations:** ^1^ Lung Cancer Center, West China Hospital, Sichuan University, Chengdu, SC, China; ^2^ Second Department of Oncology, Sichuan Friendship Hospital, Chengdu, SC, China; ^3^ Department of Abdominal Oncology, West China Hospital, Sichuan University/ West China School of Nursing, Sichuan University, Chengdu, SC, China; ^4^ Department of Abdominal Oncology, Cancer Center, West China Hospital, Sichuan University, Chengdu, SC, China; ^5^ Department of Medical Oncology, Sichuan Cancer Hospital and Institute, Sichuan Cancer Center, Medicine School of University of Electronic Science and Technology, Chengdu, SC, China

**Keywords:** apatinib, second-line treatment, hyper-vascular, biomarker, metastatic gastric cancer

## Abstract

Gastric cancer is the second most prevalent cancer and the second leading cause of cancer-related death in China. The prognosis of metastatic gastric cancer is poor with a median overall survival of 8–10 months. Apatinib, an oral small-molecule, selective vascular endothelial growth factor receptor-2 tyrosine kinase inhibitor, is approved as third-line or subsequent therapy for gastric cancer in China. Several recent small-scale studies and case reports showed that it may be great help in improvement of prognosis as second-line treatment in patients with advanced or metastatic gastric cancer. Here, we present a case of advanced gastric adenocarcinoma with multiple hepatic metastases who was treated with apatinib plus paclitaxel as second-line therapy, realized a long progression-free survival of 37 months. Until 29 January 2022, the disease remains an efficacy of partial response. We believe that the good outcome of this case is not an accident, because of the typically hyper-vascular of his liver metastases, the treatment toxicities of hypertension and proteinuria, all may be potential predictive biomarkers for anti-angiogenic treatments.

## Introduction

According to an estimation of Chinese gastric cancer from 1990 to 2019, both the incidence and mortality rank second in China (He et al., 2021). The majority of newly diagnosed cases are locally advanced or metastatic diseases. Despite many treatment options available, including chemotherapy (e.g., platinums, taxanes, and fluoropyrimidines) and targeted therapy (e.g., ramucirumab and trastuzumab), the median overall survival (OS) of advanced or metastatic gastric cancer remains a dismal 8–10 months (Shah, 2015). Ramucirumab is a human IgG1 monoclonal antibody vascular endothelial growth factor receptor-2 (VEGFR-2) antagonist. Two international phase III randomized clinical trials, REGARD and RAINBOW, showed clear efficacy benefit of ramucirumab (Fuchs et al., 2014; Wilke et al., 2014). Based on these results, National Comprehensive Cancer Network (NCCN) recommend ramucirumab as a single agent or in combination with paclitaxel as treatment options for second-line or subsequent therapy in patients with advanced or metastatic gastric adenocarcinoma. However, ramucirumab is not available in China. Apatinib, a small-molecule VEGFR-2 tyrosine kinase inhibitor (TKI), has an anticancer effect though inhibition of angiogenesis, stimulation of apoptosis, suppression of cell proliferation, and inducing the effect of conventional chemotherapy drugs (Fathi Marouf et al., 2020). Apatinib was the first anti-angiogenic drug approved for treatment of advanced or metastatic gastric adenocarcinoma by China National Medical Products Administration (NMPA) and was recommended as a third-line or subsequent therapy. Despite several studies with small sample sizes revealed that apatinib was also effective for second-line treatment of advanced gastric adenocarcinoma (Zhang et al., 2018; Yang et al., 2020; Chen et al., 2021), the treatment of apatinib as a second-line agent remains unknown. Herein, we present a metastatic gastric adenocarcinoma patient with multiple hepatic metastases, treated with apatinib as the second-line therapy and realized a long progression-free survival (PFS) of 37 months up to now (29 January 2022).

## Case description

**FIGURE 1:**
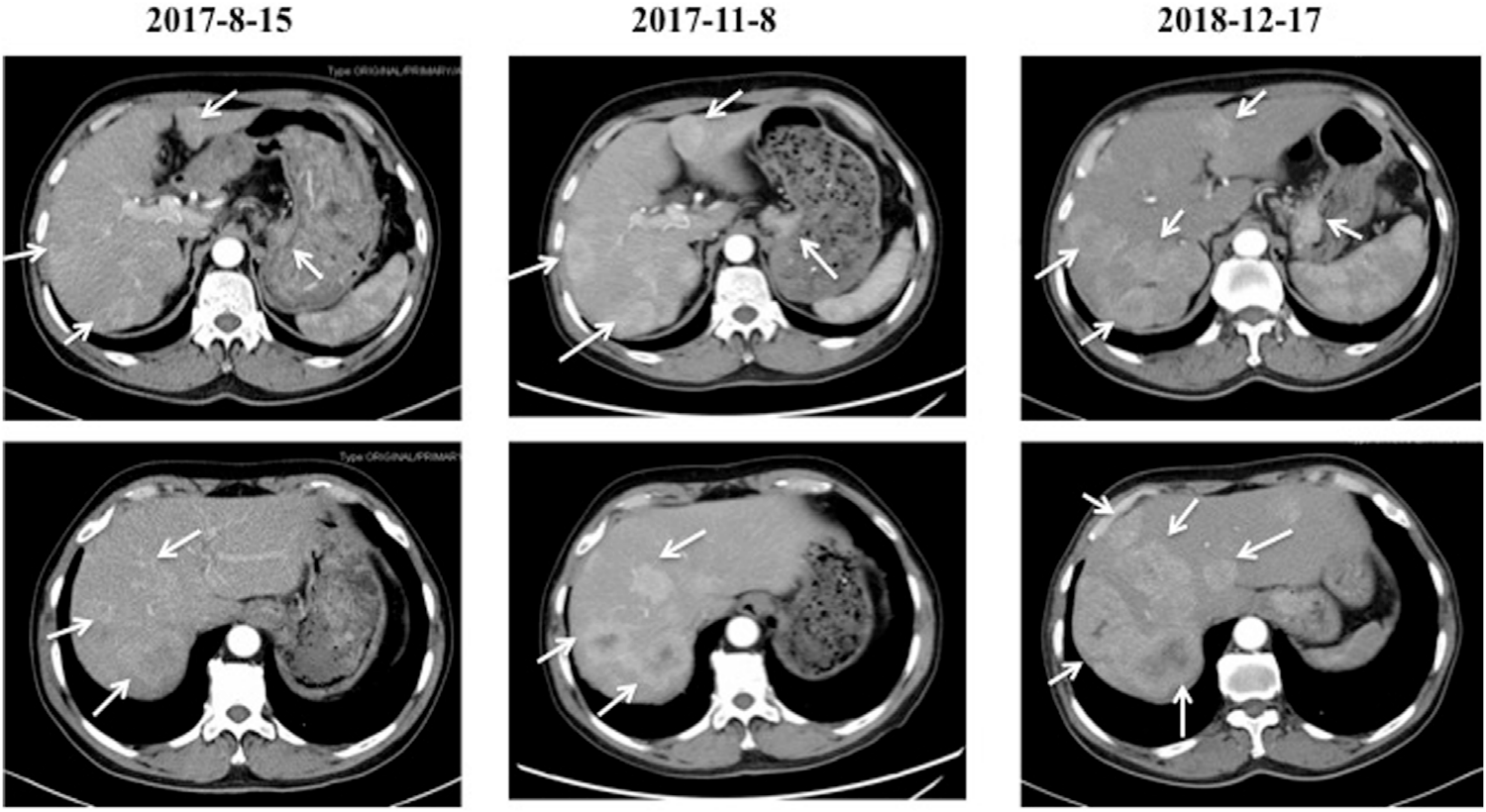
The CT images of the cardiac cancer and hepatic metastases before and after first-line chemotherapy. The cardiac tumor with multiple hepatic metastases were indicated on 15 August 2017. After two cycles of first-line chemotherapy with oxaliplatin and S-1, the cardiac and hepatic lesions showed stable disease (SD) on 8 November 2017. Five months after first-line treatment, the cardiac and liver masses were progressed on 17 December 2018.

In July 2017, a 66-year-old man attended to our hospital complaining of persistent pain in the upper abdomen for 3 years. His previous medical history was unremarkable. The thoracic and total abdomen computed tomography (CT) scan revealed gastric cardia tumor with multiple hepatic nodules. Subsequently, he underwent a gastric endoscopy and endoscopic biopsy, which revealed that primary lesion in the cardia of stomach and the mass pathology showed adenocarcinoma with negative human epidermal growth factor receptor 2 (Her-2). Based on these examinations, the patient was diagnosed with advanced gastric adenocarcinoma with multiple hepatic metastases (cT4NxM1, Stage IV). A total of eight cycles of chemotherapy with oxaliplatin and S-1 were administered. The gastric and hepatic lesions showed stable disease (SD). Five months after first-line treatment, the gastric and liver masses progressed on 17 December 2018 (Figure 1).

**FIGURE 2:**
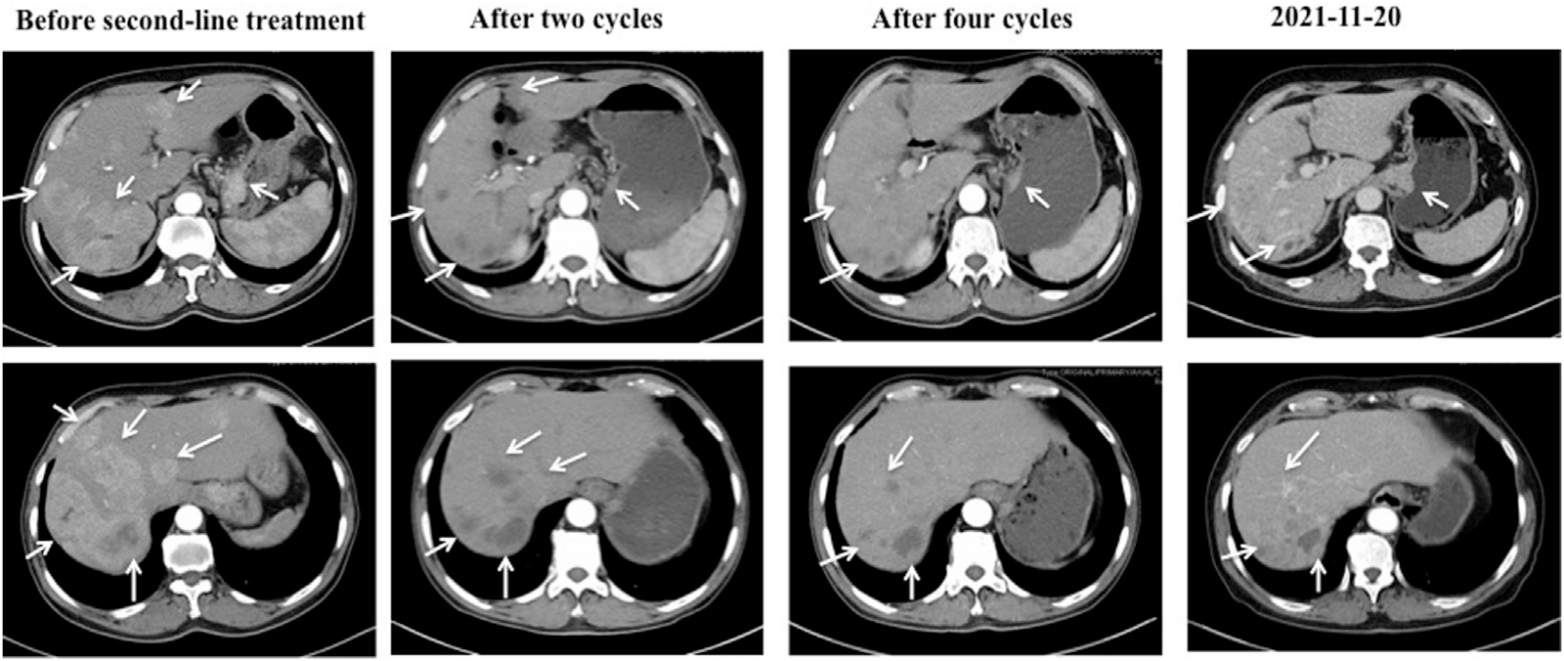
The CT images of the cardiac cancer and hepatic metastases during second-line treatment with apatinib and paclitaxel. Before second-line treatment, the CT showed markedly enlarged cardiac tumor and multiple hepatic metastases with abundant blood supply. The average CT ratio (the density of the liver metastases/the density of abdominal aorta) during arterial phase on CT imaging was 79/210. After two cycles of second-line therapy, the cardiac tumor and hepatic metastases showed a response of partial response (PR), and the ratio was 38/281 with an obviously decreasing of the degree of enhancement. Four cycles later, the efficacy remained PR. The recent CT examination was on 20 November 2021, which showed the disease remained an efficacy of PR.

**FIGURE 3:**
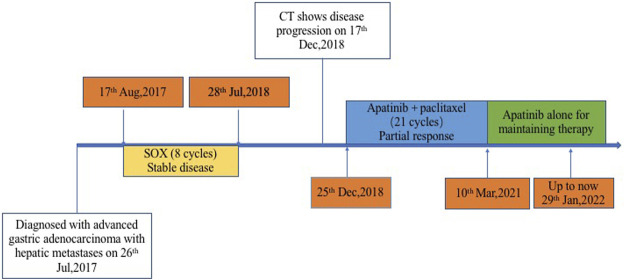
Timeline of management of this patient with metastatic gastric adenocarcinoma.

Before second-line treatment, we made comprehensive review and analysis of the dates of this case. The liver metastases of this case were typically hyper-vascular according to abdomen enhanced CT. We creatively used the average CT ratio (the density of the liver metastases/the density of abdominal aorta) during arterial phase on CT imaging to show the blood supply of liver metastasis. The ratio was 79/210 before second-line treatment. And then, considering that ramucirumab in combination with paclitaxel was recommend by NCCN as second-line treatment for patients with advanced or metastatic gastric adenocarcinoma, apatinib plus paclitaxel were selected for our case. The patient received apatinib (500 mg orally once daily) plus paclitaxel on 25 December 2018. After two cycles of therapy, the efficacy was assessed as partial response (PR). That average CT ratio was 38/281 with an obviously decreasing of the degree of enhancement (Figure 2). However, the patient developed hypertension with the highest blood pressure of 160 + mmHg, mild proteinuria (2+), and renal insufficiency with creatinine of 126 umol/L during second-line treatment. All above side effects were well controlled with appropriate treatment. The dose of apatinib was reduced to 250 mg/d on 27 April 2020. From 10 March 2021, the patient received apatinib (250 mg/d) alone for maintaining therapy (Figure 3). The recent CT examination was on 20 November 2021, which showed the disease remained an efficacy of PR. Up to now (29 January 2022), the patient demonstrated a PFS of 37 months and still received apatinib for maintaining therapy.

## Discussion

In the second-line setting of advanced or metastatic gastric cancer, chemotherapy can improve survival in patients with good performance status who are fit for chemotherapy compared with best supportive care in randomized trials. The preferred second-line chemotherapy regimens include docetaxel, paclitaxel, and irinotecan. However, the benefit from second-line chemotherapy is limited. The outcome of second-line chemotherapy in gastric cancer is extremely poor with a median OS of less than 6 months in clinical trials (Thuss-Patience et al., 2011; Kang et al., 2012; Ford et al., 2014). Ramucirumab, a VEGFR-2 antibody, has shown favorable results in two phase III clinical trials for patients with previously treated advanced or metastatic gastroesophageal cancers (Fuchs et al., 2014; Wilke et al., 2014). Based on these results, NCCN guidelines recommend ramucirumab as a single or in combination with paclitaxel as treatment options for second-line therapy in patients with advanced or metastatic gastric adenocarcinoma. However, ramucirumab is not approved by the NMPA. Apatinib, a small-molecule VEGFR-2 tyrosine kinase inhibitor (TKI), is approved as a third-line or above therapy for advanced or metastatic gastric adenocarcinoma in China according to a phase III clinical study (Li et al., 2016). Several studies also showed that apatinib is an effective regimen for the second-line treatment. Zhang et al. firstly confirmed the clinical effectiveness of second-line apatinib for advanced gastric cancer with a median PFS of 4.43 months and median OS of 9.11months (Zhang et al., 2018). In Chen’s study, compared with S-1, apatinib was superior in OS, showing a statistically significant difference (10.7 versus 8.1 months, *p* = 0.028) (Chen et al., 2021). In this report, we present a metastatic gastric adenocarcinoma received apatinib in combination with paclitaxel as second-line therapy, who had an excellent PFS of 37 months up to now. To our knowledge, this case realized the longest PFS in patients received apatinib with or without chemotherapy as second-line or above therapy.

However, there are still many advanced or metastatic gastric cancer patients failed to benefit from anti-angiogenic drugs. The high cost of anti-angiogenic treatments makes it crucial to identify biomarkers which would help select responsible patients and improving the cost to benefit ratio. Although no biomarker is fully validated for this purpose, several candidates are currently under investigation.

Circulation molecules associated with angiogenesis are the most popular potential biomarkers. The results of studies in exploring the correlation between serum VEGF/VEGFR levels and response to VEGF inhibitor treatments were complex and inconclusive. According to AVAGAST trial, high baseline plasma VEGF-A associated with a trend towards improved OS and PFS in non-Asian patients with advanced gastric cancer treated with bevacizumab (Van Cutsem et al., 2012). While no significant association was observed in Asian patients. Another analysis also identified the potential predictive value of circulating VEGF-A and VEGFR-2 in patients with metastatic breast cancer received anti-angiogenic treatment (Miles et al., 2013). And VEGF-A is being evaluated prospectively in metastatic breast cancer in the MERiDiAN trial. Serum placental growth factor (PIGF), another VEGF family member which expression levels are significantly higher in gastric cancer, also increases in response to anti-VEGF treatment (Jain et al., 2009). Soluble VEGFRs (sVEGFRs) are presented in serum as the result of alternative splicing or membrane shedding, which has a high affinity for VEGFA, and has been demonstrated to act as a naturally produced VEGF antagonist (Inoue et al., 2000). Both circulating levels of PIGF and sVEGFR were being explored as predictive biomarkers of response to anti-angiogenic drugs.

Tissue-based VEGFs have so far not been shown to be promising biomarkers for anti-angiogenic treatments. There are limitations including invasiveness and the difficulty in standardizing immunohistochemical analysis. For instance, the results obtained from tumor samples provided as slides and blocks were different. Compared with blocks, loss of immunoreactivity more often happens to paraffin-embedded tumor tissue stored on slides. Moreover, the main pitfalls of using immunohistochemistry as a quantitative measure without the consensus of standardized tests include pre-analytic tissue processing and subjective scoring.

Imaging methods are emerging as potential pharmacodynamic biomarkers because of its noninvasiveness and reproducibility. Changes in dynamic MRI-based tissue vascular measures such as blood flow, blood volume, or permeability after anti-angiogenic treatments with bevacizumab or VEGFR TKIs have been shown to occur (Schmainda et al., 2014; Kichingereder et al., 2015). Previous studies classified hepatic tumors as hyper-vascular or hypo-vascular according to the degree of contrast enhancement during the arterial phase on CT images. Hepatic tumors, which with >50% of the lesions enhances more than the adjacent liver parenchyma, are classified as hyper-vascular tumors (Katyal et al., 2000). Theoretically, hyper-vascular tumors will highly benefit from anti-angiogenic treatments. In our case, the enhanced CT disclosed that >50% of the hepatic metastases were obviously enhanced with little central necrosis. The Hounsfield unit (HU) is a relative quantitative measurement of radio density used by radiologists in the interpretation of computed tomography (CT) images. We creatively used the average CT ratio (the density of the liver metastases/the density of abdominal aorta) during arterial phase on CT imaging to show the blood supply of liver metastasis. Before second-line treatment, the average CT ratio was 79/210. Encouragingly, after two cycles of second-line treatment with apatinib plus paclitaxel, the efficacy was evaluated as PR, and the average CT ratio was 38/281 with an obviously decreasing of the degree of enhancement.

Some toxicities of anti-angiogenic drugs, including hypertension and proteinuria, can give indirect information about the outcome and might be prognostic factors. In previous studies, the rate of hypertension was consistently higher in patients treated with anti-angiogenic drugs. Anti-angiogenic drugs can reduce the production or biological activity of VEGF which is associated with decreased production of nitric oxide, causing vasoconstriction and indirectly leading to an immediate increase in blood pressure (Robinson et al., 2010). Early report suggested that the grade of hypertension might be related to the dose (Rugo et al., 2005). This point was confirmed in a meta-analysis which include seven randomized controlled trials (RCTs) (Zhu et al., 2007). Compared with low dose, high dose bevacizumab was associated with a significant increased risk of hypertension (relative risk, 7.5 versus 3.0). Whether the toxicity of hypertension correlates with better survival is controversial. Several small-scale clinical trials indicated that hypertension might be an effective biomarker and associated with a favorable OS (Khoja et al., 2014; Zhong et al., 2015). Treatment with anti-angiogenic drugs can also lead to proteinuria. A meta-analysis revealed that the summary rate of all-grade proteinuria was 13.3% among patients who were administered bevacizumab (Wu et al., 2010). Other VEGF-signaling inhibitors including apatinib and sunitinib have also been associated with proteinuria. Dose intensity was related to the incidence and the severity of proteinuria (Wu et al., 2010). Several studies attempted to explore the relationship between the incidence of proteinuria and clinical outcome but with inconsistent results. A retrospective analysis showed that proteinuria portends poorer survival in patients with metastatic colorectal cancer treated with anti-angiogenic drugs (Khoja et al., 2014). Another retrospective case series of 140 patients with recurrent glioblastoma reported converse outcome in which hypertension and proteinuria are associated with longer disease control (Carvalho et al., 2020). Our case developed hypertension, mild proteinuria, and renal insufficiency after treated with apatinib. All above side effects were well controlled with appropriate treatment. We reduced the dose of apatinib to 250 mg/d and regularly administrated blood pressure medication. After that, the patient’s blood pressure was well controlled, the degree of proteinuria ranged from negative to 1+, and the renal function kept normal. Up to now (29 January 2022), this patient has maintained continuous PR.

After reviewing the previous published literatures about apatinib as second-line or above therapy in patients with advanced or metastatic gastric cancer, we are sure that the present case in our report realized the longest PFS. We believe that the encouraging outcome of our case is not accident. The typically hyper-vascular hepatic metastases, treatment toxicities of hyertension and proteimuria in this patient may be potential predictive biomarkers for anti-angiogenic tretment. In second-line therapy, apatinib combination with chemotherapy might be an alternative treatment for some selected advanced or metastatic gastric adenocarcinoma. Further well-designed prospective clinical studies are necessary to explore the efficacy of apatinib alone or combined with chemotherapy as a second-line treatment and the predictive biomarkers of apatinib in advanced or metastatic gastric cancer.

## Data Availability

The original contributions presented in the study are included in the article/supplementary material, further inquiries can be directed to the corresponding author.
